# Prediction of Permeability and Efflux Using Multitask Learning

**DOI:** 10.1021/acsomega.5c04861

**Published:** 2025-11-05

**Authors:** Philip Ivers Ohlsson, Gian Marco Ghiandoni, Susanne Winiwarter, Rocío Mercado, Vigneshwari Subramanian

**Affiliations:** † Department of Computer Science and Engineering, 11248 Chalmers University of Technology and University of Gothenburg, Chalmersplatsen 1, 412 96 Gothenburg, Sweden; ‡ Chemical Toxicology, Clinical Pharmacology and Safety Sciences, Biopharmaceuticals R&D, AstraZeneca, Pepparedsleden 1, 431 83 Mölndal, Sweden; § Augmented DMTA Platform, Data Analytics and AI, The Discovery Centre (DISC), R&D IT, AstraZeneca, Francis Crick Avenue, Cambridge CB2 0AA, U.K.; ∥ Drug Metabolism and Pharmacokinetics, Research and Early Development, Cardiovascular, Renal and Metabolism (CVRM), BioPharmaceuticals R&D, AstraZeneca, Pepparedsleden 1, Mölndal 431 83, Sweden

## Abstract

*In silico* prediction of cell membrane permeability is crucial in drug discovery, since a compound’s permeation through membranes influences parameters such as its in vivo efficacy, bioavailability, and pharmacokinetics. This study investigates the use of multitask graph neural networks to predict a selection of permeability-related endpoints. The study utilized a harmonized, single-laboratory internal data set of over 10K compounds measured in human colorectal adenocarcinoma (Caco-2) and Madin–Darby canine kidney (MDCK) cell lines, routinely employed in experimental assays for drug permeability and efflux. This data set is an order of magnitude larger than comparable public collections, thus providing greater statistical power and a consistent error profile for model development. A series of multitask learning (MTL) models trained on such data were benchmarked against single-task approaches and evaluated on an external public data set to investigate the model’s applicability domain. The comparison between the performance of single- and multitask models suggests that MTL can achieve higher accuracy by leveraging shared information across endpoints. MTL is also shown to perform better when augmented with molecular features. In particular, the inclusion of *p*K_a_ and LogD, is shown to improve the accuracy of both permeability and efflux endpoints. This work presents benchmarking results of models utilizing different data splitting strategies, accompanied by guidelines for optimal validation in the context of MTL.

## Graphical abstract

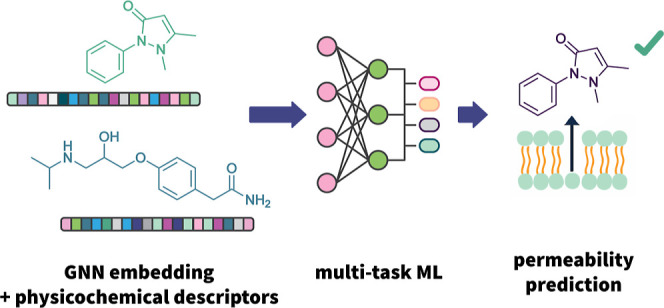

## Introduction

Permeation across cell membranes is an important factor influencing drug disposition, its pharmacokinetic profile and its in vivo efficacy; hence, permeability assessment is considered critical from the early stages of the discovery process.^1–3^ Experimental data on permeability has been generated for many years utilizing various cell types, such as the Caco-2 or MDCK-cell lines.^2^ The Caco-2 cell line^4^ was originally derived from human colon carcinoma cells and is used extensively in pharmaceutical research to assess intestinal permeability, captured by determining the apparent permeability, P_app_, across the cell monolayer. P_app_ is a measurement of how quickly a substance that has been placed on one side of a membrane appears on the other, and is measured in the absorptive, or apical-to-basolateral (a-b), direction. In addition, the presence of intestinal efflux transporters such as P-glycoprotein (P-gp), breast cancer resistance protein (BCRP), and multidrug resistance protein 1 (MRP1) in Caco-2 cells allows the estimation of both active and passive permeation. A compound’s potential to be an efflux substrate is quantified by determining the efflux ratio (ER), based on measurements of apparent permeability in both apical-to-basolateral and basolateral-to-apical directions (ER = P_app_ (b-a)/P_app_ (a-b)). Similarly, the MDCK cell line,^5^ derived from canine kidney cells with very low native transporter activity,^6^ is a useful tool to capture efflux ratios. The MDCK cell line was shown to be well-suited to transfection with human transporter genes such as MDR1, i.e., the gene encoding the human P-gp transporter which serves as a gatekeeper, e.g., in the blood–brain barrier. The ER determined in such transfected MDCK-MDR1 cells gives specific information about a compound’s interactions with P-gp and helps assess a compound’s likelihood to enter the brain.

Permeability data has been used frequently to develop in silico models, from simple regression models^7–9^ to more sophisticated machine learning (ML) architectures,.^10–16^ Wang et al.^10^ investigated parametric and nonparametric methods for predicting Caco-2 P_app_, reporting gradient boosting as their best-performing model. Wang and Chen^12^ reported dual radial basis function neural networks as their best model for predicting Caco-2 P_app_, showing metrics comparable to those of Wang et al.^10^ Lanevskij and Didziapetris^13^ proposed linear regression and least-squares methods for the prediction of Caco-2 P_app_, enabling the possibility for interpretation and thereby gaining mechanistic insights. Geylan et al.^16^ investigated tree-based methods and support vector machines for the prediction of cyclic peptide permeabilities measured in four different cell lines, concluding that ML predictors may fail to perform accurately beyond their applicability domain and suggesting an approach for extrapolation. Finally, Fang et al.^14^ investigated the prediction of various ADME properties, including MDR1-MDCK ER, using a variety of ML approaches and molecular representations, and concluded that message-passing neural networks (MPNNs) performed best across all endpoints.

In addition to these single-task permeability models, the use of MTL was reported in Feinberg et al.,^17^ where permeability was modeled as one of the endpoints together with multiple other endpoints.^15^ The authors showed that a PotentialNet^18^ multitask model trained on 31 ADMET assays led to improved permeability predictions when compared to single-task random forest models trained on fingerprints.^17^ Yet another study^19^ highlights the use of MTL for predicting MDR1 and BCRP efflux activities, concluding that the ensemble ML approach on descriptors and graphs offers higher predictive performance. In a recent study by Peteani et al.,^15^ MTL was used to predict P_app_ from MDCK, PAMPA, and Caco-2 assays, and ER from an MDCK-MDR1 assay of targeted protein degraders.

These reports show that reliable permeability estimations can be achieved by in silico *predictions* and that such predicted data are valuable in the drug discovery process. However, available experimental data is often scarce, fragmented across different assay formats, and strongly condition-dependent, making it difficult to assemble consistent training sets. In addition, much of the existing permeability data is locked in proprietary sets and cannot be shared publicly. On the modeling side, passive permeability is mainly governed by physicochemical properties such as lipophilicity, size, polarity, and ionization, whereas active transport also depends on specific compound–transporter interactions, which are more complex to capture computationally. These factors limit the development of accurate and publicly accessible permeability prediction models.

To the best of our knowledge, the use of MTL with added features to predict permeabilities and efflux ratios across all modalities and the relevance of different splitting strategies for multitask model evaluations have not been discussed elsewhere exhaustively. Herein, we compare single-task learning (STL) and MTL to predict cell permeability and efflux endpoints using a collection of curated data from AstraZeneca’s first-in-line screening assays. For the assessment of intestinal absorption, Caco-2 P_app_ (a-b) is measured in the presence of transport inhibitors to obtain passive (intrinsic) permeability across the membrane using a pH gradient to mimic the intestinal environment.^2^ For efflux, several options are used in-house. ERs are measured in both Caco-2 and MDCK-MDR1 cell lines. In these assays, neither transporter inhibitors nor a pH gradient are present. In addition, internal efflux data were included in the study utilizing the more sensitive MDCK-MDR1 cell line of the National Institutes of Health (NIH),^20^ NIH MDCK-MDR1. We used the data to train and evaluate a series of MPNN models using Chemprop^21^ in single- and multitask settings. In addition, we included a series of baseline random forest regressors in the benchmark as a comparison between MPNNs and a classical ML approach. Furthermore, we explored the effect of different data splitting strategies and assessed the possibility to augment Chemprop by including precalculated molecular features as input.

Our analysis shows how cross-learning can leverage data from distinct Caco-2 and MDCK-MDR1 assays, thus improving the accuracy of models in an MTL setting. We conclude that multitask MPNNs augmented with predicted LogD and *p*K_a_ as additional descriptors outperform the other evaluated methods across the permeability and efflux endpoints investigated in our study. Further assessment of our models’ generalization capabilities shows that the feature-augmented MPNN can yield reliable predictions for different chemical modalities (macrocycles, peptides, and PROTACs) with performance metrics similar to those for small molecules, which make up the bulk of the training data. Finally, this publication includes the artifact file of the MPNN model that does not require additional features (GNN-MTL), retrained with a newer version of Chemprop, which can be used by the community as-is or fine-tuned using transfer learning.

## Materials and Methods

### Data

#### Internal Data Collection

For this study, we constructed an aggregated data set by combining AstraZeneca’s internal data for four permeability-related endpoints. In particular, we collected data for passive (intrinsic) permeability measured in Caco-2 cells and efflux data determined in Caco-2, MDCK-MDR1 (referred to as MDCK) and NIH MDCK-MDR1 (referred to as NIH MDCK) cell lines (Figure 1a). The assays are described in the following subsections.

**Figure 1:**
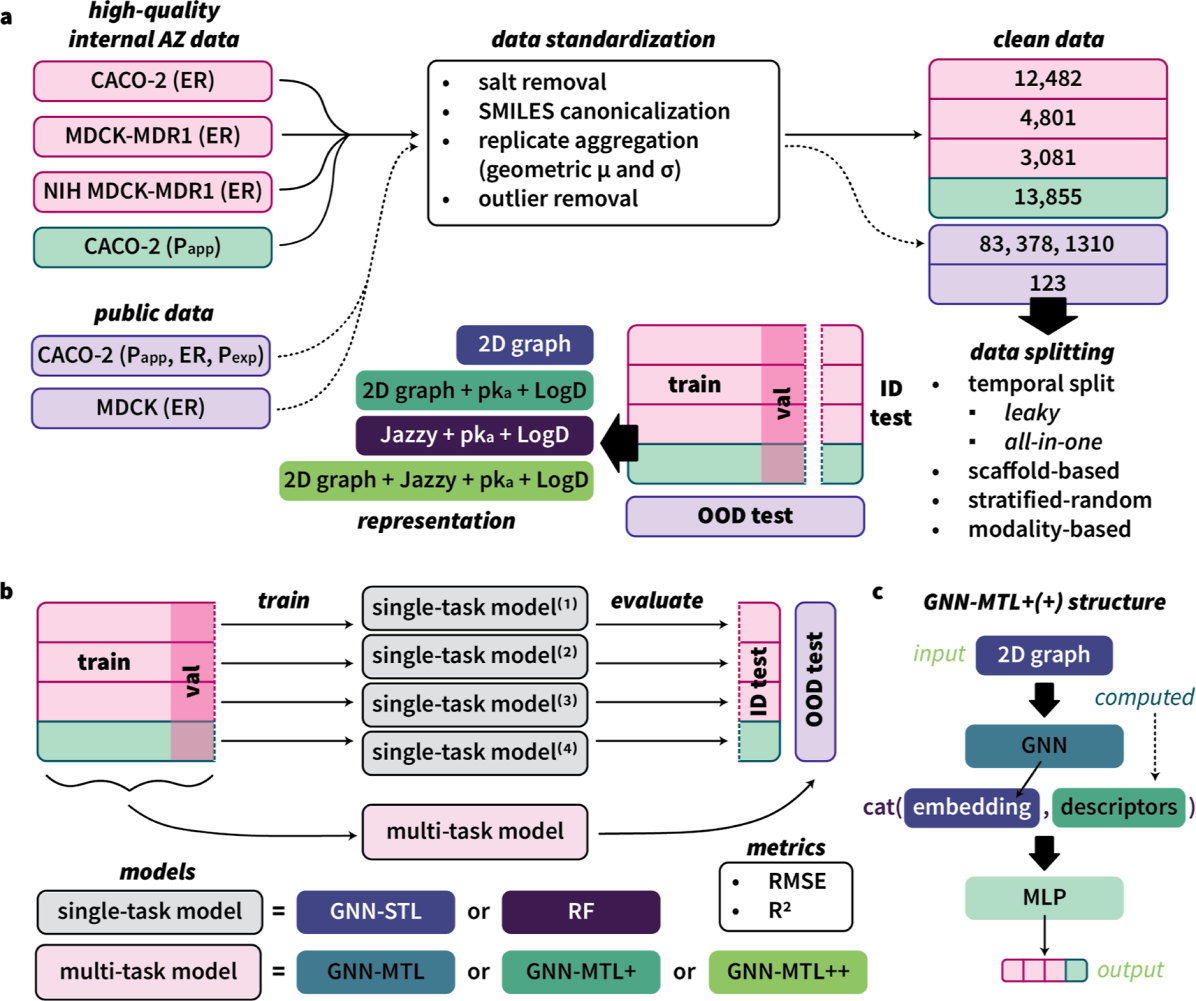
(a) Data processing pipeline. ID test: in-distribution test set; OOD test: out-of-distribution test set. Internal data were sourced from AstraZeneca (AZ). Public data were sourced from NMMPDB^22^ and CycPeptMPDB.^23^ (b) Model training and evaluation pipeline. GNN: graph neural network; RF: random forest; STL: single-task learning; MTL: multitask learning; MLP: multilayer perceptron. GNN-MTL+: multitask graph neural network with additional *p*K_*a*_ and LogD features; GNN-MTL++: multitask graph neural network with additional *p*K_a_, LogD, and RDKit features. (c) In the case of the GNN-MTL+ and GNN-MTL++ models, the learned graph embedding generated by the GNN from an input 2D graph is concatenated with additional descriptors (GNN-MTL+: *p*K_*a*_ + LogD; GNN-MTL++: *p*K_*a*_ + LogD + RDKit features) before being passed into an MLP to generate the predictions (output).

#### Intrinsic Caco-2 Permeability Assay

The permeation rate of a compound across the Caco-2 cell monolayer is measured in an automated assay setup, in the presence of inhibitors of the main efflux transporters, P-gp, BCRP, and MRP1, as described by Fredlund et al.^2^ The procedure is carried out by adding a given compound to the apical side of the cell layer. The concentrations on both the apical and basolateral sides are then measured after 45 and 120 min and both the permeation rate (apparent permeability, P_app_, expressed in 1 × 10^–6^ cm/s) and recovery are determined. On the apical side, a pH of 6.5 is maintained, whereas on the basolateral side, the pH is kept at 7.4, mimicking the pH gradient seen in the small intestine.^2^

#### Efflux Assays

ER is measured in an automated assay setup, very similar to the one described above.^2^ Permeability is measured in both directions by adding a compound on either the apical or basolateral side of the cell layer and measuring concentrations on both sides after 2 h. ER is calculated as P_app_ (b-a) divided by P_app_ (a-b). The pH is kept at 7.4 on both sides, and no inhibitors are used. ER data from three different cell lines were used in this study: Caco-2, MDCK-MDR1, and NIH MDCK-MDR1 cell lines. Caco-2 contains several human transporters (P-gp, BCRP, and MRP1), thus it gives information on whether a compound may be a substrate of any of these. In contrast, the MDCK-MDR1 and NIH MDCK-MDR1 cell lines are MDCK cell lines transfected with human MDR1, thus only expressing P-gp. These assays are used to determine whether a compound may specifically be a P-gp substrate.

#### Standardisation and Outlier Removal

For all compounds in the data set, SMILES strings were standardized using the ChEMBL structure pipeline package in Python.^24^ In the data set, there were a few values considered as out-of-bound measurements, i.e., measurements exceeding the quantifiable range of the method, usually indicated as “lower than X” or “greater than X”. Those values were retained as such after excluding the < and > qualifiers. All measurements were converted to logarithmic scale and aggregated by SMILES; the mean log value for each compound was calculated, provided there were multiple measurements. In addition to mean values, standard deviations (σ) were calculated to quantify experimental errors.

To account for the heteroscedasticity in the data, we estimated the σ_pred_ of individual predictions based on the trends in the experimental data. A linear regression model was fit against the mean logarithmic values (independent variable) and standard deviations (dependent variable) as described by Wenlock and Carlsson.^25^ The model was used to identify outliers in the data by comparing their σ_calc_ and σ_pred_. Compounds with σ_pred_ that exceeded 2σ_calc_ were labeled as outliers and removed. The data standardization pipeline is summarized in Figure 1a.

From the data cleaning pipeline, the following data sets were obtained: Caco-2 ER and Caco-2 P_app_ contained 12,482 and 13,855 points, respectively; MDCK and NIH MDCK contained 4801 and 3081 points, respectively. The distributions of values for Caco-2 ER and P_app_ are reported in Figure 2 and MDCK and NIH MDCK ER in Figure 3. The data sets were aggregated to form a multiendpoint data set of 22,907 unique SMILES, where 1% entries had data for all 4 endpoints, 5% of entries had data for 3 endpoints, 37% of entries had data for 2 endpoints, and 57% of entries had only 1 endpoint. In the cleaned data set, standard deviations for most compounds for which multiple measurements exist were <0.1 or 0.2 for all endpoints, indicating that experimental values are usually closer than within 2-fold. More specifically, for efflux endpoints >90% of the compounds with multiple measurements showed a standard deviation < 0.1, whereas this was the case for only just above 50% of the compounds with multiple permeability measurements. However, >75% of the permeability data show a standard deviation below 0.2. Heteroscedasticity is clearly seen for Caco-2 P_app_ (Figure 2), with higher variability for lower values, which is in line with the experimental setup. Very low concentrations are expected for low permeability values and these are often difficult to measure. On the other hand, heteroscedasticity is less obvious in the case of efflux measurements, although one would assume that compounds with high ER values show higher variability as high efflux is often caused by low permeation in the *a* to *b* direction. In general, we anticipate that the variability for compounds with singleton data would be similar to that of compounds with multiple measurements.

**Figure 2:**
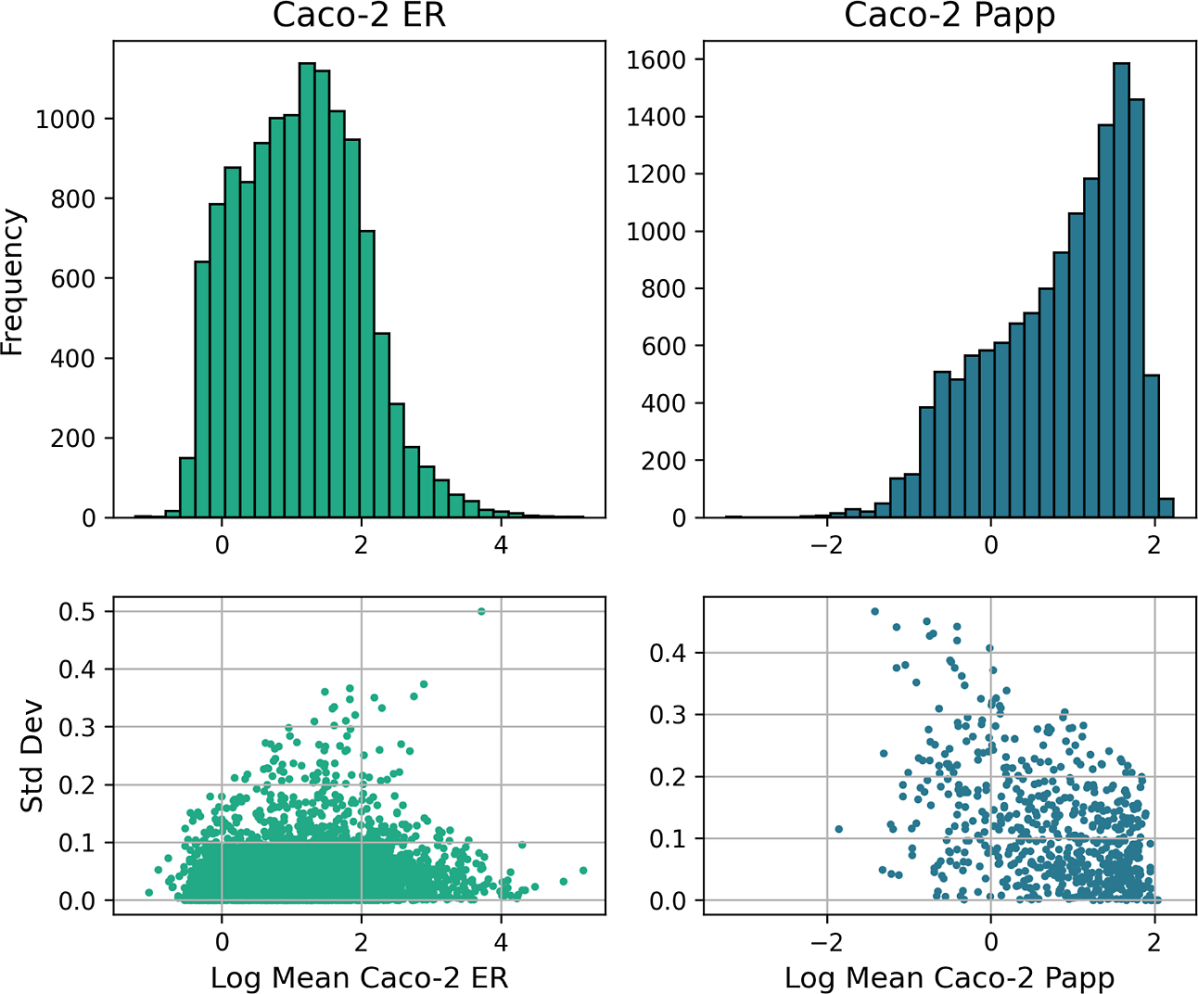
Left and right histograms report the data distributions for Caco-2 LogER and LogP_app_, respectively. Caco-2 ER measurements appear to be approximately normally distributed, centered between 1 and 2. A tapering effect is observed on the right tail, extending up to around 4–5. Caco-2 P_app_ shows a right-skewed distribution, with a peak occurring at values between 1 and 2.

**Figure 3:**
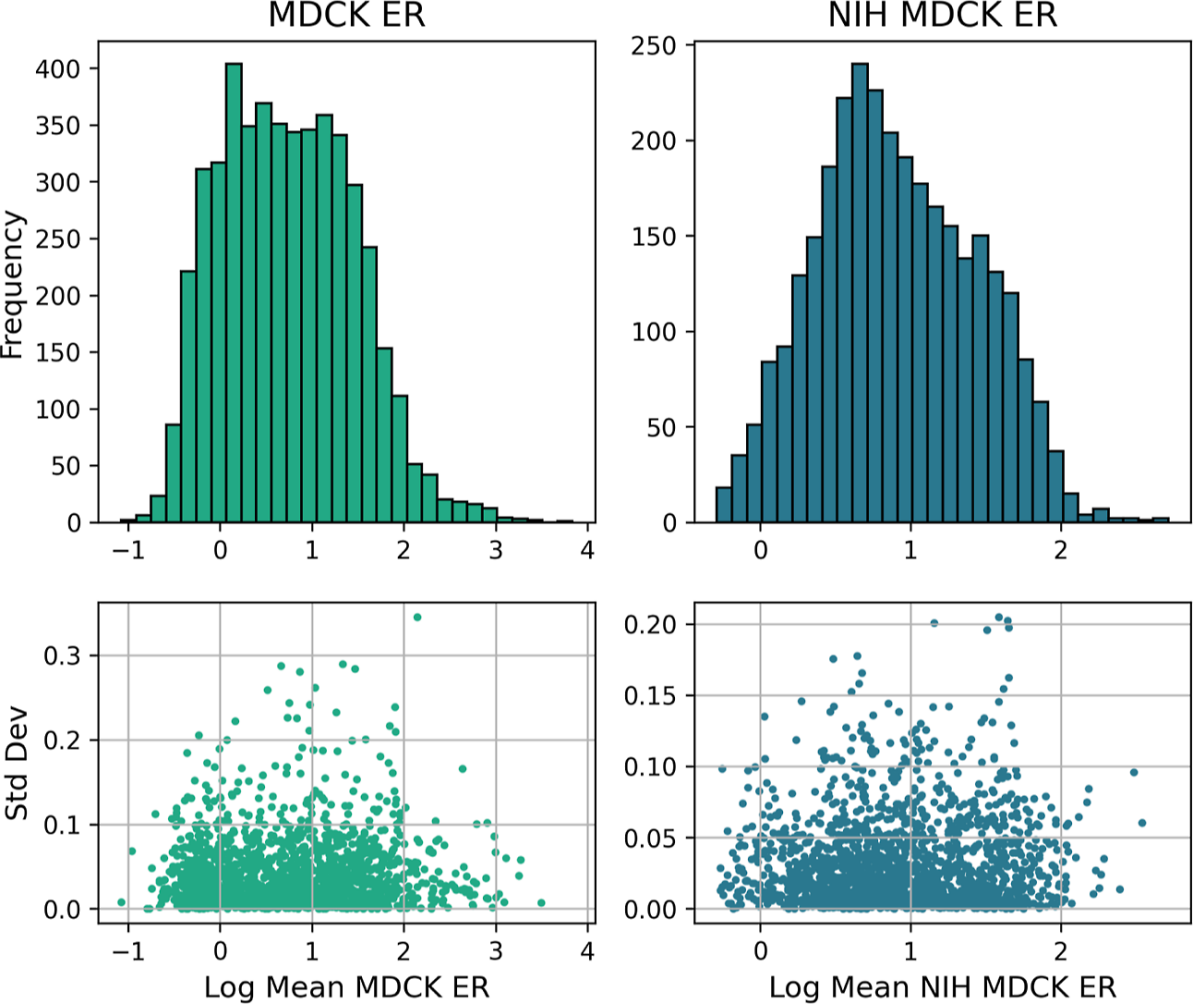
Left and right histograms report the data distributions for MDCK LogER and NIH MDCK LogER, respectively. Both data distributions appear to be approximately normal and peak between 0 and 1. Both distributions also show a slight tailing on the right beyond values of 2.

#### Data Splitting

Several data-splitting strategies were employed to evaluate the performance and applicability domains of the models (Figure 1a). Specifically, we considered stratified-random, scaffold-balanced, modality-based, and temporal splitting approaches.

In the *stratified-random* splitting strategy, training and test sets were generated by selecting molecules at random while ensuring that the distribution across endpoints remained the same. In our case, 20% of the data set was randomly designated as the test set while enforcing stratification. To improve the robustness of the validation, we applied it to five train-test splits and averaged their metrics. This is also referred to as 5-split repeated holdout validation.

The validation was performed by calculating the root mean squared error (RMSE) between predictions and experimental values of the compounds in the test sets for each endpoint. RMSE was chosen over other validation metrics due to its better interpretability, sensitivity to large errors, and to ease the comparison between the performance of the model across different test sets. Therefore, RMSE was also adopted for the additional validations presented as follows.

To assess the ability of models to predict novel chemical structures, we applied *scaffold-based* splitting, which groups compounds according to their Bemis and Murcko scaffolds.^26^ In this approach, training and test sets were generated to ensure no overlap between scaffold types. Data partitioning followed an 80:20 ratio, consistent with the stratified-random strategy. To achieve balanced split sizes, molecules were iteratively assigned to the least populated split based on their scaffold structure, a process referred to as core-based stratification. Five training-test splits (i.e., 5-split repeated holdout validation) were evaluated and their metrics averaged as for the previous splitting strategy.

To evaluate predictive generalizability across distinct chemical modalities, including macrocycles, peptides, PROTACs, and small molecules, we assessed the best model obtained from the previous validations (GNN-MTL++; see Results and Discussion) using *modality-based* splitting. Each compound was categorized into a modality type using predefined internal annotations. This validation was conducted using a 5-fold cross-validation (CV), where accuracy was evaluated across different modality groups.

Finally, our best model (GNN-MTL++; see Results and Discussion) was also assessed using *temporal* splitting. In our implementation of temporal splitting, the data are partitioned using timestamps, with the validation being initiated on a smaller training set and progressively incorporating test data. This approach quantifies how learning improves as more data become available over time. Here, temporal splitting was implemented using two strategies: *leaky* and *all-for-one*. *Leaky* splitting, designed for multitask problems, allows compounds to appear in both training and test sets but for different endpoints. For instance, a compound with data for Endpoint A and Endpoint B could be used for training with Endpoint A and tested on Endpoint B. While the model does not explicitly learn to predict Endpoint B, the cross-learning capabilities of MTL may enhance its predictions, making this validation “leaky.” In contrast, *all-for-one* enforces a stricter rule: if a compound appears in the test set for any endpoint, it is excluded from the training set entirely. This prevents cross-learning but at the cost of reduced data availability for evaluation.

In this work, both *leaky* and *all-for-one* temporal splits are implemented by first sorting all data points according to their timestamp, from oldest to newest based on the acquisition date. The training and test sets are then generated from the sorted data by partitioning the data into chunks to allow five rounds of validation, as shown in Tables 1 and 2. For instance, in case of Caco endpoints, ∼15% of the data was used for training and ∼85% for testing in the first round, ∼32% went for training and ∼68% for testing in the second round, and so on. The fifth and last round included around 80% of the data in the training set and 20% in the test set. In the *all-for-one* scenario, standardized SMILES were used as a reference to ensure that any SMILES present in the test set would not be present in the training set.

**Table 1:** Distribution of Test Set Percentages and Compound Overlap for Caco-2 P_app_ and Caco-2 ER Across Different Splits Using *leaky* Temporal Splitting^a^

split	Caco-2 P_app_ (%)	training overlap (%)	Caco-2 ER (%)	training overlap (%)
1	84.71	0.62	85.63	1.66
2	66.07	0.71	66.57	1.44
3	48.61	1.68	48.79	2.04
4	36.57	3.11	35.94	1.49
5	19.98	2.65	19.65	1.08

a Test set percentages represent the proportion of test compounds relative to the total number of compounds per endpoint, while overlap percentages indicate the proportion of test compounds that also appear in the overall training set.

**Table 2:** Distribution of Test Set Percentages and Compound Overlap for MDCK ER and NIH MDCK ER Across Different Splits Using *leaky* Temporal Splitting^a^

split	MDCK ER (%)	training overlap (%)	NIH MDCK ER (%)	training overlap (%)
1	67.28	1.07	100.00	0.77
2	46.28	2.03	100.00	1.85
3	21.70	2.53	100.00	7.64
4	12.96	4.89	51.83	3.97
5	4.89	9.27	20.71	5.90

a Test set percentages represent the proportion of test compounds relative to the total number of compounds per endpoint, while overlap percentages indicate the proportion of test compounds that also appear in the overall training set.

The entire data sets created for the *leaky* and *all-for-one* evaluation were additionally validated using 5-fold CV (80:20 ratio) to compare their results with those from the stratified-random and scaffold-balanced splitting validation.

#### External Test Set

To further validate and investigate the applicability domain of the best model (GNN-MTL++; see Results and Discussion), we retrained the model on all internal data and used external data sets sourced from CycPeptMPDB^23^ v1.2 (accessed Dec 27, 2024) and NMMPDB^22^ (accessed Jan 11, 2025). Initially, we extracted 1332 Caco-2 *P*_exp_ values from CycPeptMPDB. Further, 83 Caco-2 P_app_, 383 Caco-2 ER, and 123 MDCK ER values were gathered from NMMPDB. The permeabilities extracted from CycPeptMPDB were represented as *P*_exp_ and expressed in 1 × 10^–6^ cm/s, which is an unstandardised form of the apparent permeability P_app_ as values are aggregated from multiple distinct publications. Compounds whose permeability values were not within the detectable limits were assigned a value of −10 × 10^–10^ cm/s in CycPeptMPDB, and *P*_exp_ values corresponding to those compounds were considered outliers and removed, which in turn resulted in 1310 Caco-2 *P*_exp_ values. In the case of Caco-2 P_app_, Caco-2 ER, and MDCK ER, all values were within the recommended range and no outliers were detected.

### Models

The model training and evaluation pipeline is summarized in Figure 1b.

#### Baseline

We trained a series of random forest (RF) regressors from scikit-learn^27^ as a baseline model. All the models were trained on individual data sets and hence no data leakage or cross-learning is expected. Our RF models were trained using 145 RDKit descriptors (see Supporting Information) and seven Jazzy descriptors.^28,29^ However, the evaluation of Jazzy descriptors was discontinued due to a significant proportion of compounds failing the 3D embedding preprocessing step required to calculate their partial charges.

#### Single-Task GNN

We evaluated graph neural networks (GNNs) for permeability prediction in a single-task setting. Given the four different endpoints, we trained four distinct GNN models using Chemprop^21^ v1.7.0, where each model returns a single endpoint (scalar) as output; we denote these models as GNN-STL. The GNN-STL models were validated using data sets from the individual endpoints such that no data aggregation was needed for STL. Hence, no effect from leaking or cross-learning is expected for these models, as that of the baseline RF regressors.

#### Multitask GNN

We also evaluated the GNN implementation in Chemprop in a multitask setting by training a single multitask model, GNN-MTL, to predict a four-dimensional vector corresponding to the four endpoints in the data. The GNN-MTL model was evaluated using data sets containing the aggregated data of all endpoints.

Two additional GNN-MTL models were trained by including molecular descriptors in the assessment (Figure 1c). Chemprop provides an option to include descriptors by concatenating them to the learned MPNN embedding and passing them into a multilayer perceptron (MLP) that ultimately generates the predictions (Figure 1a). The first model, referred to as GNN-MTL+, was trained by incorporating LogD and *p*K_a_ along with the graph representation; these two physicochemical properties were computed using internal AZ models and were selected due to their well-established role in modulating permeability.^30,31^ The second model, referred to as GNN-MTL++, was trained by extending the LogD and *p*K_a_ descriptors to include a selection of RDKit descriptors, namely PEOE_VSA1, MolWt, SlogP_VSA1, qed, TPSA, BertzCT, NHOHCount, and RingCount; more information on these descriptors can be found in the official RDKit documentation.^32^ These descriptors were selected by applying the SHAP^33^ method to our RF model trained using RDKit descriptors. Only the top 5 descriptors relevant for predicting each endpoint were taken into consideration (Figure S3).

## Results

### Evaluation on Internal Data

#### Design Strategies for Temporal Data Splitting

Data splitting for multitask validation can be challenging to visualize due to the presence of multiendpoint data within the same set. To clarify the composition of training and test sets across different temporal splits, we provide a detailed breakdown in the following tables. Table 1 contains the test set percentages (relative to the total number of compounds for each endpoint) and the overlap between training and test sets for Caco-2 P_app_ and Caco-2 ER across *leaky* temporal splits. Similarly, Table 2 reports these metrics for MDCK ER and NIH MDCK ER.

As shown in Table 1, the overlap between test and training sets was minimal for Caco-2 P_app_ (2.65%) and Caco-2 ER (1.08%). Table 2 indicates a higher degree of overlap for MDCK ER (9.27%) and NIH MDCK ER (5.90%). This difference is likely due to the larger size of the Caco-2 data set (13,855 and 12,482 data points) compared to MDCK (4801 and 3081 data points), leading to increased leakage from the Caco-2 data when validating MDCK models. Additionally, Table 2 shows that, for NIH MDCK ER, the test set contained 100% of the compounds in the first three splits, implying that no NIH MDCK ER data was included in the training set during these iterations. This absence of training data is a consequence of the NIH MDCK ER assays being introduced later at AstraZeneca, when compared to other endpoints.

In contrast to the leaky splitting discussed above, the *all-for-one* splitting approach ensures that no compounds in the test set are present in the training set. Despite being a stringent validation strategy, there is a potential downside to this approach that results in reduced test set sizes with the progression of folds. For instance, less than 6% and 1% of the data are in the fifth split of the test sets of Caco-2 P_app_ and NIH MDCK ER, respectively (Table 3). The reduction in test set size can lead to high variability in performance metrics, making it difficult to draw statistically significant conclusions while validating smaller splits. The increase in performance variability due to the decreasing size of test sets, coupled with the high structural variability across scaffolds expected from temporal splitting, constitute the main challenges of this validation strategy. These observations highlight the need for careful design of temporal validation frameworks in MTL.

**Table 3:** Percentage Distribution of Test Sets Across Different Splits for Each Endpoint Using *all-for-one* Temporal Splitting^a^

split	Caco-2 P_app_ (%)	Caco-2 ER (%)	MDCK ER (%)	NIH MDCK ER (%)
1	78.86	82.37	95.21	76.57
2	62.32	69.65	83.05	25.58
3	45.01	55.69	62.11	3.38
4	26.09	39.54	45.62	1.56
5	6.83	23.56	27.91	0.81

a This illustrates how test set sizes decrease as more data are progressively allocated to training in the *all-for-one* split scenario.

#### Repeated Holdout and Cross Validation

To evaluate the model’s robustness, we implemented a repeated hold-out validation for stratified-random and scaffold-balanced splits using a 80:20 splitting strategy and captured the variations in performances based on five splits. On the other hand, for the temporal *leaky* and *all-for-one* splits, we performed a 5-fold CV to estimate the variability in prediction performances (see Materials and Methods for detailed information about the splits). The outcomes of these validations corresponding to the five different models (RF, GNN-STL, GNN-MTL, GNN-MTL+, and GNN-MTL++) are exemplified in terms of RMSEs and their respective variabilities are indicated by the error bars in Figure 4.

**Figure 4:**
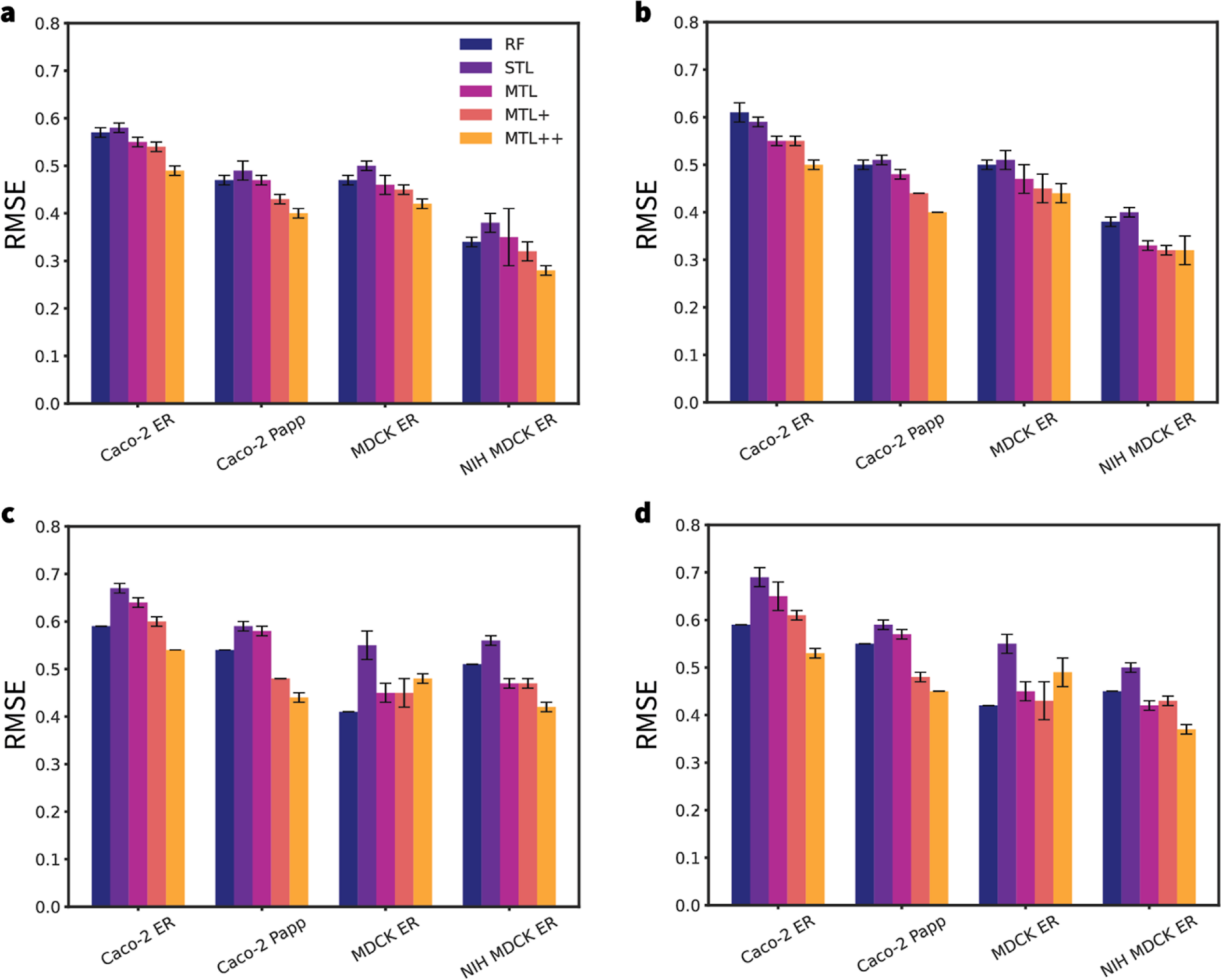
RMSE comparison across various endpoints for the RF, GNN-STL (STL), GNN-MTL (MTL), GNN-MTL++ (MTL+), and GNN-MTL++ (MTL++) models, assessed using 5-split validation for (a) the *stratified-random* split, (b) the *scaffold-balanced* split, and 5-fold CV for (c) the *leaky* split, and (d) the *all-for-one* split. Bars and error bars represent the mean RMSE and standard deviation in the test set results across different splits and folds. The legend in the first panel applies to all bar charts.

The results of the *stratified-random* split evaluation (Figure 4a; see Tables S1 and S2 in the Supporting Information for details) show similar RMSE values for the RF, GNN-STL, GNN-MTL, and GNN-MTL + models. The GNN-MTL++ model, on the other hand, produced significantly better metrics for all endpoints, especially when compared to those from RF and STL, suggesting improved accuracy due to the inclusion of additional descriptors. Similarly, in the *scaffold-balanced* split evaluation (Figure 4b; see Tables S1 and S2 in the Supporting Information for details), the GNN-MTL++ model consistently outperforms the RF and STL models, implying improved generalizability across different chemical motifs. These results demonstrate that incorporating additional molecular descriptors related to permeability can be leveraged by the underlying Chemprop model.

The cross-validation results for the *leaky* (Figure 4c; see Tables S1 and S2 in the Supporting Information for details) and *all-for-one* (Figure 4d; see Tables S1 and S2 in the Supporting Information for details) data sets exhibit slightly different trends. In some cases, the RF regressor performs comparably to or better than the Chemprop models. However, the metrics still indicate that GNN-MTL++ is the best-performing model across all endpoints. The only exception is the performance of GNN-MTL++ on MDCK ER, where it appears to perform worse for the *leaky* and *all-for-one* settings. The GNN-MTL++ performance drop in the *leaky* split can be attributed to a slight overfit introduced by the selected RDKit descriptors that fail to generalize well for MDCK ER. In case of the *all-for-one* splitting, the comparatively higher RMSE values of GNN-MTL++ could indicate that MDCK ER is more sensitive to the data partitioning when the selected RDKit descriptors are used. These results show that MTL generally performs better than RF and STL, possibly due to its cross-learning capabilities.

#### Temporal Validation

Figure 5a,b illustrate the variation in RMSE values for the *leaky* and *all-for-one* validation splits using GNN-MTL++, the best-performing model according to the results from the validations presented previously. Both *leaky* and *all-for-one* produced similar RMSE values overall (refer to Tables 1, 2, and 3 to infer the test set proportions for the different splits). An expected key trend, particularly for Caco-2 ER and P_app_, is that errors are higher in the earlier splits, where less training data are available and larger test sets are evaluated. The error decreases as more training examples are incorporated from the test sets. However, in some cases, RMSE initially declines but then increases in later splits before dropping again, suggesting that simply adding more data does not always improve performance. This variability is more pronounced in the *all-for-one* results, where no data leakage occurs.

**Figure 5:**
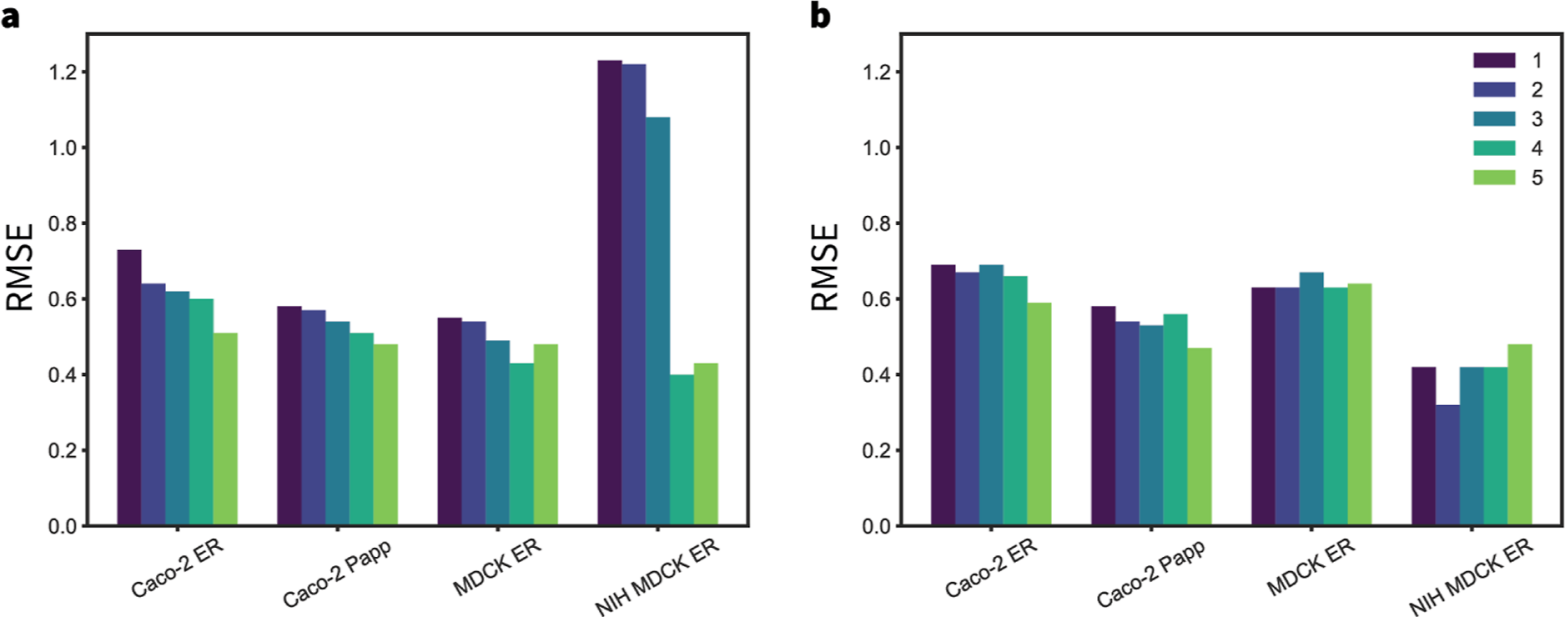
RMSE comparison across various endpoints for each split of the temporal validation for (a) the *leaky* split and (b) the *all-for-one* split. The legend in the second panel applies to both bar charts. Note how cross-learning enables a decrease in the RMSE of NIH MDCK ER across the first three splits despite the model having seen no training examples for this endpoint.

The comparison between *leaky* and *all-for-one* trends highlights the cross-learning capabilities of Chemprop. Notably, for NIH MDCK ER (Table 2), no training examples were included in the first three *leaky* splits. As such, the variations in RMSE values across these folds must be attributed solely to cross-learning from other endpoints, demonstrating this as a key advantage of MTL.

### Evaluation of Model Performance Across Different Modalities

Figure 6 shows the performance of the GNN-MTL++ model across different endpoints and molecular modalities—including macrocycles, peptides, PROTACs, and small molecules—using *modality-based* splitting. Performance is most accurate for small molecules compared to other modalities; generally, lower RMSE values are observed for modalities with a greater number of data points (Figure 6b; see Table S3 in the Supporting Information for details). In particular, the MDCK and NIH MDCK studies include very small sample sizes for some categories of modalities; for instance, macrocycles for NIH MDCK endpoint have as few as one example. Limited data availability for macrocycles and peptides makes it challenging to reach reliable conclusions regarding the performance estimates of MDCK and NIH MDCK ER.

**Figure 6:**
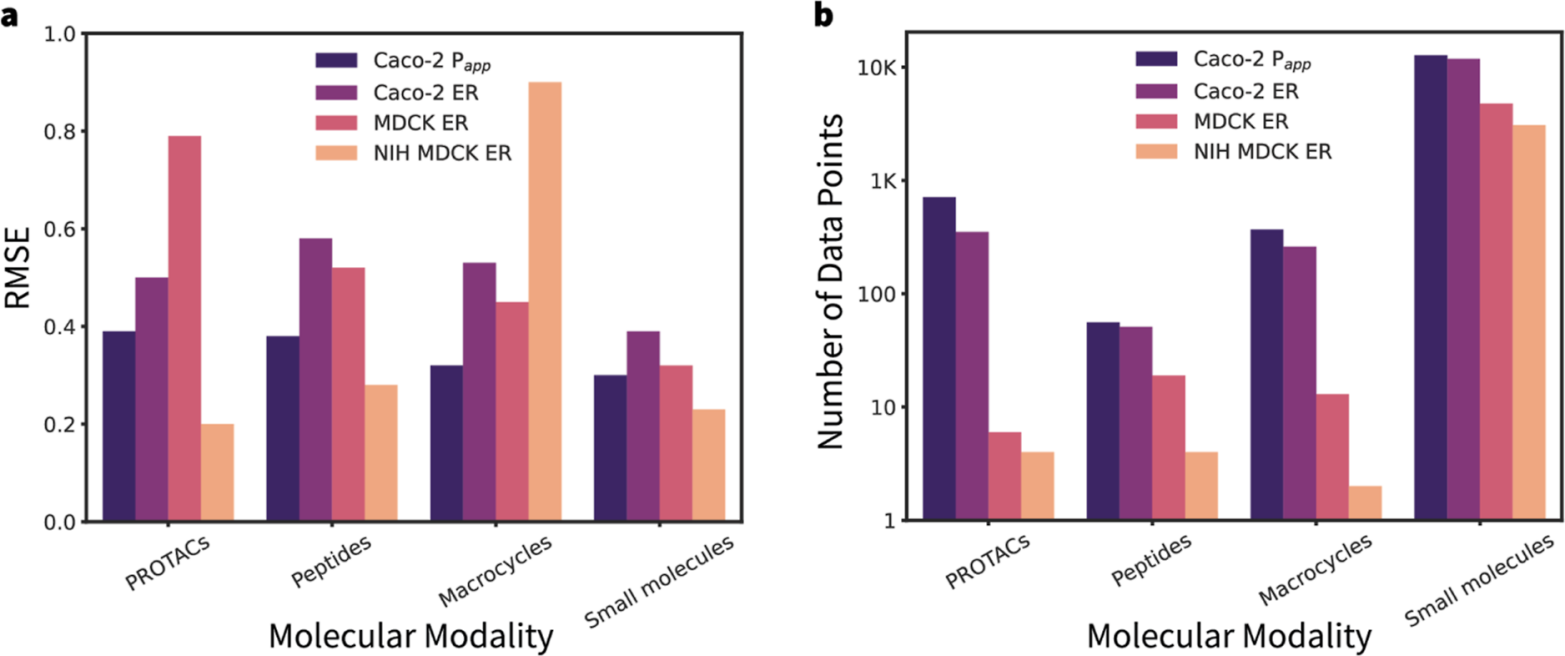
(a) RMSE comparison of the GNN-MTL++ model predictions across the following four molecular categories: PROTACs, peptides, macrocycles, and small molecules. (b) Number of data points per endpoint and modality, illustrating that way more data were available for small molecules than any other modality. Bar plots in both panels are colored according to the endpoints.

### Evaluation on External Test Sets

To externally validate the performance of the GNN-MTL++ model, we assessed its predictive accuracy using publicly available Caco-2 and MDCK experimental permeability assay data from NMMPDB^22^ and CycPeptMPDB.^23^ Parity plots comparing the model predictions to experimental values are shown in Figure 7.

**Figure 7:**
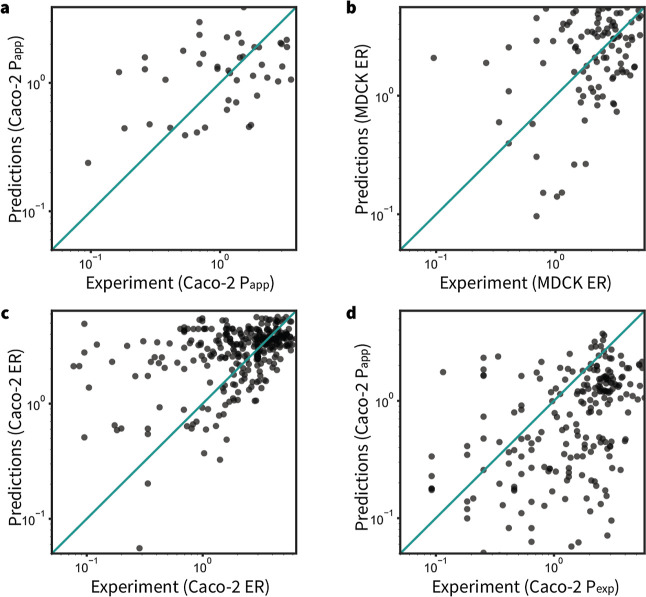
Comparison of predictions made by the GNN-MTL++ model against public experimental data. (a) Predicted Caco-2 P_app_ versus Caco-2 P_app_ values from NMMPDB (ρ = 0.61). (b) Predicted MDCK ER versus MDCK ER values from NMMPDB (ρ = 0.42). (c) Predicted Caco-2 ER versus Caco-2 ER values from NMMPDB (ρ = 0.50). (d) Predicted Caco-2 P_app_ versus Caco-2 *P*_exp_ values from CycPeptMPDB^23^ (ρ = 0.29). ρ: Spearman’s rank correlation coefficient. All values are log-scaled.

Overall, the model exhibits a strong correlation with experimental data, despite the inherent variability introduced by differences in assay conditions and data sources. This external validation is particularly challenging, as the test data are heterogeneous, i.e., aggregated from multiple sources with varying degrees of (unknown) systematic and instrumental error, and heavily biased toward peptide and macrocycle permeability assays, of which there are fewer than 400 examples per endpoint in the internal data set. Consequently, we consider these data sets true out-of-distribution (OOD) test sets. Given their heterogeneity and potential noise, the Spearman’s rank correlation coefficient (ρ) was employed as a more robust metric to evaluate the qualitative agreement between predictions and experimental values.

Figure 7a illustrates the GNN-MTL++ model’s predictions for Caco-2 apparent permeability (P_app_) compared to experimental values (P_app_, × 10^–6^ cm/s) from NMMPDB. The model achieves a Spearman’s correlation of ρ = 0.61 and an RMSE of 2.64, suggesting it captures overall permeability trends well, though individual predictions may vary in magnitude. Figure 7b compares the MDCK ER predictions against the experimental ER values from NMMPDB, yielding ρ = 0.42 and an RMSE of 1.70, indicating moderate predictive performance. In Figure 7c, the model’s predictions for Caco-2 ER are evaluated against experimental values from NMMPDB, resulting in ρ = 0.50 and an RMSE of 1.75 and thus reflecting a reasonable, though not highly accurate, alignment with experimental data. Finally, Figure 7d compares predicted Caco-2 P_app_ values with experimental permeability data (*P*_exp_, × 10^–6^ cm/s) from CycPeptMPDB. Here, the model achieves a weaker correlation of ρ = 0.29 and an RMSE of 1.69, suggesting greater challenges in accurately predicting permeability in this data set. Overall, while the GNN-MTL++ model effectively captures general permeability trends, its predictive accuracy varies across different data sets and endpoints, being notably better once again for Caco-2 than MDCK endpoint predictions. Once again, this is likely due to the greater number of Caco-2 data points present in the training data (Figure 6b).

For comparison, Supporting Information Figure S4 presents results for the GNN-MTL model, which uses no additional features besides the molecular graph representation, on the public data set. Note that the GNN-MTL achieves a higher average RMSE across the three endpoints (Caco-2 P_app_, Caco-2 ER, and MDCK ER) and a significantly lower mean Spearman correlation (ρ_GNN‑MTL++_ = 0.46 vs ρ_GNN‑MTL_ = 0.27). This further supports our observations from the internal data validations and reinforces the idea that incorporating additional features such as *p*K_a_, LogD, and RDKit descriptors enhances the model’s predictive performance.

## Discussion

We have benchmarked a series of models to predict four permeability and efflux endpoints considering two different scenarios: (1) applying cross-validation, repeated holdout, and temporal validation on internal data; and (2) validating on public data, which we consider a far OOD test set. Among the internal validations, our implementation of temporal splitting offered insights on the capabilities of models to deal with unseen data prospectively, and allowed quantification of learning and cross-learning for incremental amounts of training data.

### Experimental Variability and Its Impact on Model Performance

Our results highlight that, as expected, evaluating model performance on external data presents greater challenges. The external data sets comprise permeability and efflux measurements from two public databases that aggregate data for cyclic peptides and other macrocycles. Although these compound classes are represented in the in-house data set, the majority of the internal data consists of small molecules, making the chemical space of the public data distinct. Furthermore, the experimental conditions in the public data sets might not be consistent with the internal data, as neither database specifies whether their data were generated under a pH gradient or in the presence of inhibitors. It is highly probable that the public data are derived under a variety of conditions.

Our in-house permeability measurements were obtained using Caco-2 cells in the absorptive (apical-to-basolateral, *a*-*b*) direction, with a pH gradient to mimic intestinal conditions (pH 6.5 on the apical side and pH 7.4 on the basolateral side). These assays were conducted in the presence of an inhibitor cocktail to minimize the influence of efflux transporters, yielding higher permeability measurements. In summary, our model’s tendency to overestimate the permeability of the test molecules when predicting Caco-2 P_app_ in the NMMPDB data might simply be attributable to divergent experimental conditions. Assuming that at least some of the measurements in NMMPDB performed without the presence of transport inhibitors, lower permeability values due to transporter-mediated efflux would be expected in the external data.

Similarly, experimental variability is likely in the ER data, too. Our ER measurements were conducted without a pH gradient and without inhibitors, which is assumed to be the standard setup for such assays. However, it is possible that ER measurements are obtained using transporter inhibitors for specific investigations. In addition, the MDCK cell lines used in our study were transfected with the human MDR1 gene, but efflux measurements in MDCK cells can also be performed using nontransfected cells, and several transfected MDCK cell-lines with varying sensitivity exist. As no details on the exact cell lines or experimental procedures are recorded, some inconsistencies are expected.

As we point out possible sources of variability in the public data, we believe that it is worth restating that the conditions of our assays are defined and consistent for each measurement, and these must be taken into account by the readers of this manuscript and users of the GNN-MTL model that we are releasing. The variability of our in-house data sets was assessed during the data curation process (Figures 2 and 3) and was generally low across all endpoints, with standard deviations for compounds with multiple measurements typically <0.1 on a logarithmic scale. The Caco-2 P_app_ permeability distribution followed a different distribution, with a typical standard deviation < 0.2 and a somewhat greater variability for measurements below 1 (LogP_app_ < 0). So even if data is generated under standardized experimental conditions, we see variability. This suggests that external data sets, which aggregate measurements from multiple sources, are likely to exhibit even greater inconsistencies (see Supporting Information Figures S1 and S2) due to differences in experimental protocols, assay conditions, and measurement techniques.

### Challenges in Predicting External Data

These discrepancies help explain why our model showed weaker generalization to public data sets. While absolute permeability predictions were sometimes off by an order of magnitude, the best model consistently preserved the relative rankings of endpoints, achieving Spearman’s rank correlation coefficients >0.4 across all P_app_ and ER endpoints. Our results highlight the challenges of cross-data set generalization, but also demonstrate the model’s ability to capture meaningful trends despite the significant differences between internal and external data.

We acknowledge the difficulty in directly benchmarking our model performance against previously published permeability or efflux models, since assay conditions, chemical spaces, and data-splitting strategies vary widely in the literature. Therefore, our study intentionally focused on a controlled evaluation of multitask learning within a consistent experimental and computational framework, rather than systematic comparisons across model architectures. Nevertheless, based on the reported performance metrics from recent permeability studies,^14,15^ our results appear consistent with state-of-the-art models.

### Generalisation Capabilities on New Modalities

Despite the challenges in applying our models to heterogeneous external data, the GNN-MTL++ model showed strong generalization to modalities such as PROTACs, peptides, and macrocycles, achieving RMSE values < 1.0 across all endpoints. This is particularly noteworthy given that these modalities are under-represented in the training data compared to small molecules. The model’s ability to generalize to less explored regions of chemical space suggests that MTL, coupled with informative physicochemical descriptors such as LogD and *p*K_a_, enhances predictive robustness. However, integrating features generated by separate machine learning submodels comes with a caveat that such a reliance introduces additional variability, particularly when evaluating compounds that fall outside the applicability domain of these submodels. We believe that advancing predictive performance for currently under-represented modalities requires the acquisition of additional experimental data, a process that may be enhanced by leveraging foundation models.

## Conclusion

In this study, we demonstrated that multitask learning (MTL) outperforms both single-task learning (STL) and random forest (RF) baselines for molecular permeability and efflux predictions. The most pronounced improvements are observed for data-sparse endpoints, which can likely be attributed to cross-learning among related tasks. Enhancing GNN-MTL with additional molecular features (GNN-MTL+ and GNN-MTL++) led to further performance gains; however, we observed that incorporating external features can introduce overfitting, as seen with GNN-MTL++ in the leaky MDCK ER split. Evaluation across random, scaffold, and temporal data splits highlighted the inherent challenges posed by heterogeneous, asynchronously generated data sets (e.g., compound overlap, small test sets). External validation was constrained by variable assay conditions in public data sets, which may introduce bias depending on differences from the experimental setup used for training. As part of this work, we retrained the baseline GNN-MTL model using the latest Chemprop version and are releasing it for public use (either as-is or for further fine-tuning). This contribution is intended to provide a practical tool and valuable guidance for molecular permeability prediction in cheminformatics and drug discovery.

## Supplementary Material



## Data Availability

We make the following data available with this work: The artifact file of the GNN-MTL model, retrained using Chemprop v2.1.0, and not requiring additional features from submodels, is available at the following DOI doi.org/10.5281/zenodo.16948542. The model generates four output columns in the following order “Caco2ER”, “Caco2Papp”, “MDCKER”, “NIHMDCKER”. Models were trained on internal AstraZeneca data collected up to May 5, 2024. This data are proprietary and have not been made publicly available. Public data from CycPeptMPDB^23^ is available at http://cycpeptmpdb.com/ and was downloaded Jan 2025, corresponding to v1.2; public data from NMMPDB^22^ is available at https://swemacrocycledb.com/ and was downloaded Jan 2025. We used Chemprop v1.7.0 for all MPNN models constructed in this work, available at https://github.com/chemprop/chemprop.

## Notes

Additional details are available in the Supporting Information PDF, including: description of RDKit descriptors used, SHAP feature importance plots for random forest models, data distributions for the public data, public data predictions with the GNN-MTL model, and additional tables of results (PDF)
