# Supplementary material for: Prediction of Permeability and Efflux Using Multitask Learning
Source: ACS Omega. 2025 Nov 5;10(45):54148–59. doi: 10.1021/acsomega.5c04861 (PMC12631307; doi:10.1021/acsomega.5c04861)
Supplement: Supplementary file 1 [file ao5c04861_si_001.pdf]

Prediction of permeability and efflux using multi-task learning

Philip Ivers Ohlsson,[†,‡,‖] Gian Marco Ghiandoni,[¶,‖] Susanne Winiwarter,[§] Rocío Mercado,[*,†] and Vigneshwari Subramanian[*,‡]

†*Department of Computer Science and Engineering, Chalmers University of Technology and University of Gothenburg, Chalmersplatsen 1, 412 96 Gothenburg, Sweden*

‡*Chemical Toxicology, Clinical Pharmacology and Safety Sciences, Biopharmaceuticals R&D, AstraZeneca, Pepparedsleden 1, 431 83 Mölndal, Sweden*

¶*Augmented DMTA Platform, Data Analytics and AI, R&D IT, AstraZeneca, The Discovery Centre (DISC), Francis Crick Avenue, Cambridge CB2 0AA, United Kingdom*

§*Drug Metabolism and Pharmacokinetics, Research and Early Development, Cardiovascular, Renal and Metabolism (CVRM), BioPharmaceuticals R&D, AstraZeneca, Pepparedsleden 1, 431 83 Mölndal, Sweden*

‖*These authors contributed equally.*

E-mail: rocio.mercado@chalmers.se; vigneshwari.subramanian@astrazeneca.com

RDKit Descriptors

The following RDKit descriptors were included in the GNN-MTL+ and GNN-MTL++ models:

MaxAbsEStateIndex	MinAbsEStateIndex	MinEStateIndex
qed	SPS	MolWt
MaxPartialCharge	MinPartialCharge	MaxAbsPartialCharge
FpDensityMorgan1	FpDensityMorgan2	FpDensityMorgan3
BCUT2D_MWHI	BCUT2D_MWLOW	BCUT2D_CHGHI
BCUT2D_CHGLO	BCUT2D_LOGPHI	BCUT2D_LOGPLOW
BCUT2D_MRHI	BCUT2D_MRLOW	AvgIpc
BalabanJ	BertzCT	HallKierAlpha
Ipc	PEOE_VSA1	PEOE_VSA10
PEOE_VSA11	PEOE_VSA12	PEOE_VSA13
PEOE_VSA14	PEOE_VSA2	PEOE_VSA3
PEOE_VSA4	PEOE_VSA5	PEOE_VSA6
PEOE_VSA7	PEOE_VSA8	PEOE_VSA9
SMR_VSA1	SMR_VSA10	SMR_VSA2
SMR_VSA3	SMR_VSA4	SMR_VSA5
SMR_VSA6	SMR_VSA7	SMR_VSA9
SlogP_VSA1	SlogP_VSA10	SlogP_VSA11
SlogP_VSA12	SlogP_VSA2	SlogP_VSA3
SlogP_VSA4	SlogP_VSA5	SlogP_VSA6
SlogP_VSA7	SlogP_VSA8	TPSA
EState_VSA1	EState_VSA10	EState_VSA11
EState_VSA2	EState_VSA3	EState_VSA4
EState_VSA5	EState_VSA6	EState_VSA7
EState_VSA8	EState_VSA9	VSA_EState1
VSA_EState10	VSA_EState2	VSA_EState3

VSA_EState4	VSA_EState5	VSA_EState6
VSA_EState7	VSA_EState8	VSA_EState9
FractionCSP3	NHOHCount	NOCount
NumAliphaticCarbocycles	NumAliphaticHeterocycles	NumAliphaticRings
NumAromaticCarbocycles	NumAromaticHeterocycles	NumAromaticRings
NumHAcceptors	NumHeteroatoms	NumRotatableBonds
NumSaturatedCarbocycles	NumSaturatedHeterocycles	NumSaturatedRings
RingCount	MolLogP	

Data Distributions For The Public Data

In Figure S1 we compare the endpoint distribution from the NMMPDB database with the distribution in predictions for the same molecules using two of the MTL models developed herein, illustrating distributions using kernel density estimates (KDEs). In Figure S2 we plot the distributions of the endpoints collected from CycPeptMPDB for using KDEs.

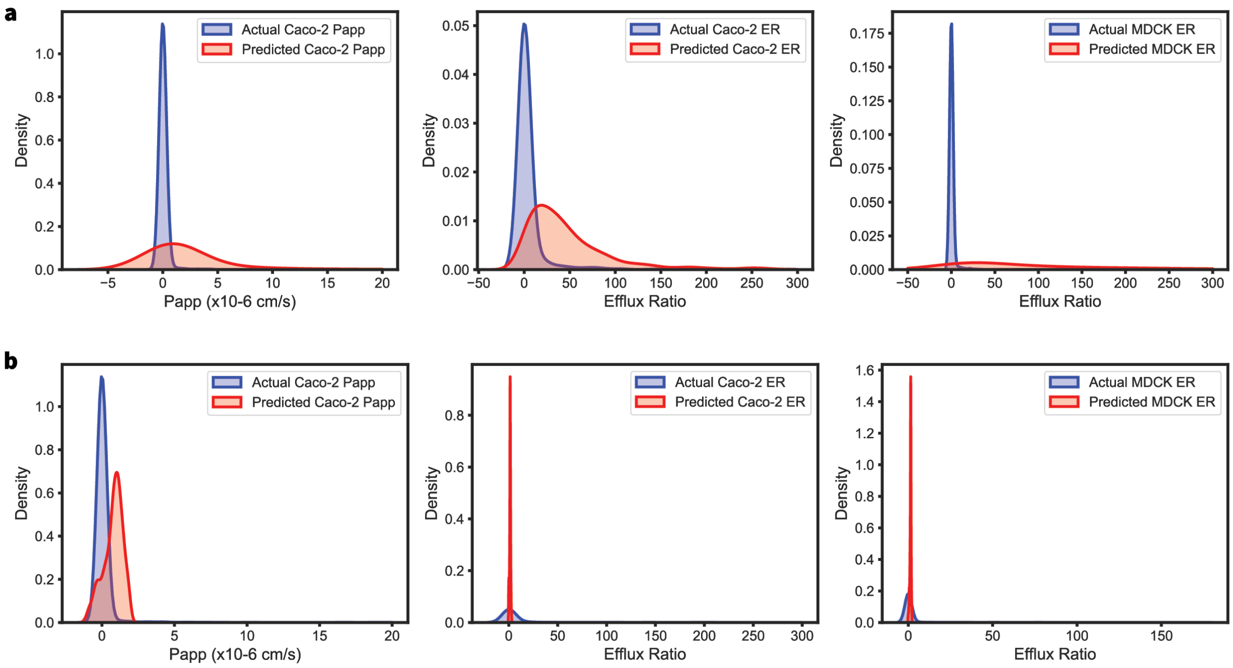

Figure S1: a) Comparison of the endpoint distribution (KDEs) from NMMPDB (blue) with the distribution in the predicted values for the same molecules using the GNN-MTL++ model (red). b) Comparison of the same experimental data distributions (blue) with the distribution in the predicted values for the same molecules using the GNN-MTL model (red).

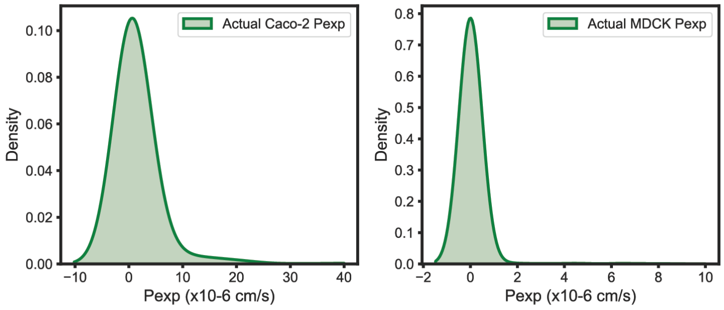

Figure S2: KDEs of the values in the CycPeptMPDB database for Caco-2 *(left)* and MDCK *(right)* assays.

SHAP Feature Importance Plots For Random Forest Models

In Figure S3 we show the feature importance plots for the individual random forest models trained on four different end points using RDKit descriptors. The top 5 features selected from each plot were used as additional descriptors to train the GNN-MTL++ models.

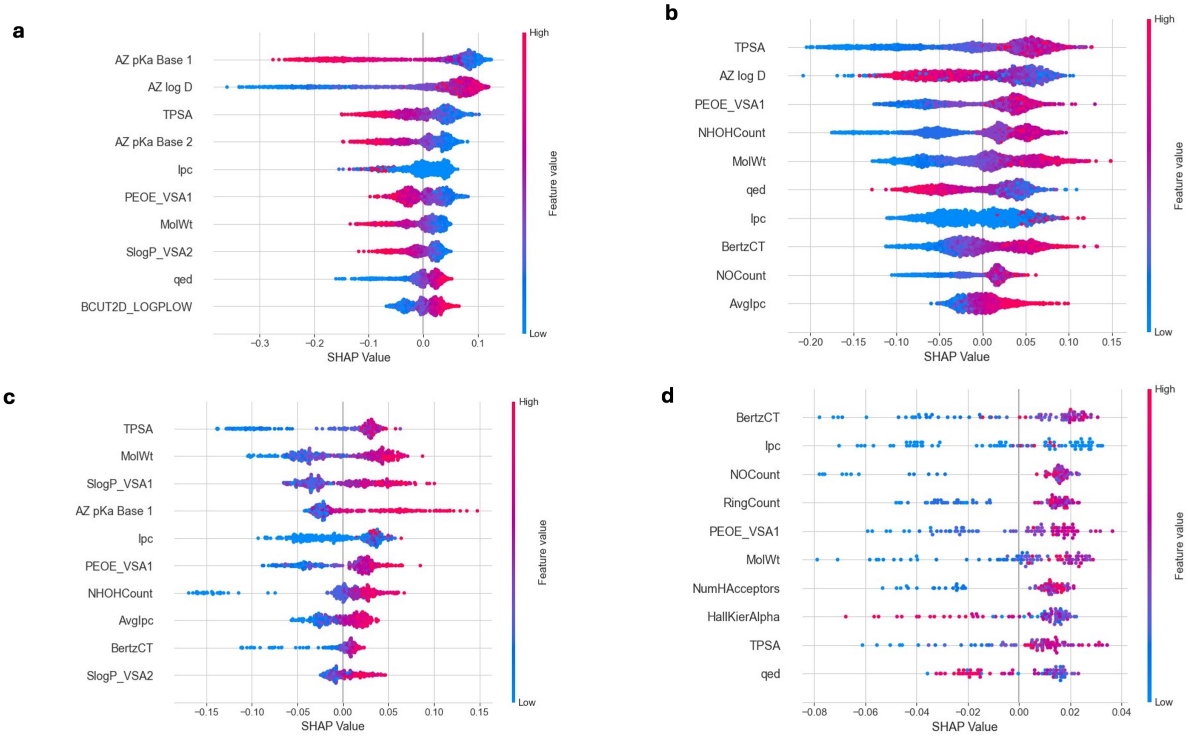

Figure S3: Feature importance plots generated by applying SHAP to the random forest models trained with RDKit descriptors. a) Caco-2 ER, b) Caco-2 , c) MDCK ER, and d) NIH MDCK ER.

Public Data Predictions With The GNN-MTL Model

In Figure S4 we demonstrate the performance of the GNN-MTL model with no additional descriptors. As can be seen, the absolute predictions with the GNN-MTL model are slightly worse than those of the GNN-MTL++ model (mean RMSE across the three Caco-2 , Caco-2 ER, and MDCK ER endpoints: $RMSE_{GNN-MTL}$= 8.58; $RMSE_{GNN-MTL++}$= 1.87), and the Spearman rank correlation coefficients are significantly worse for the GNN-MTL model, suggesting that the additional descriptors (i.e., , LogD, and Jazzy descriptors) greatly im-

prove the predictive capabilities of the model on public data (mean Spearman's rank coefficient across the three Caco-2 , Caco-2 ER, and MDCK ER endpoints: $\rho_{GNN-MTL}$= 0.27; $\rho_{GNN-MTL++}$= 0.46).

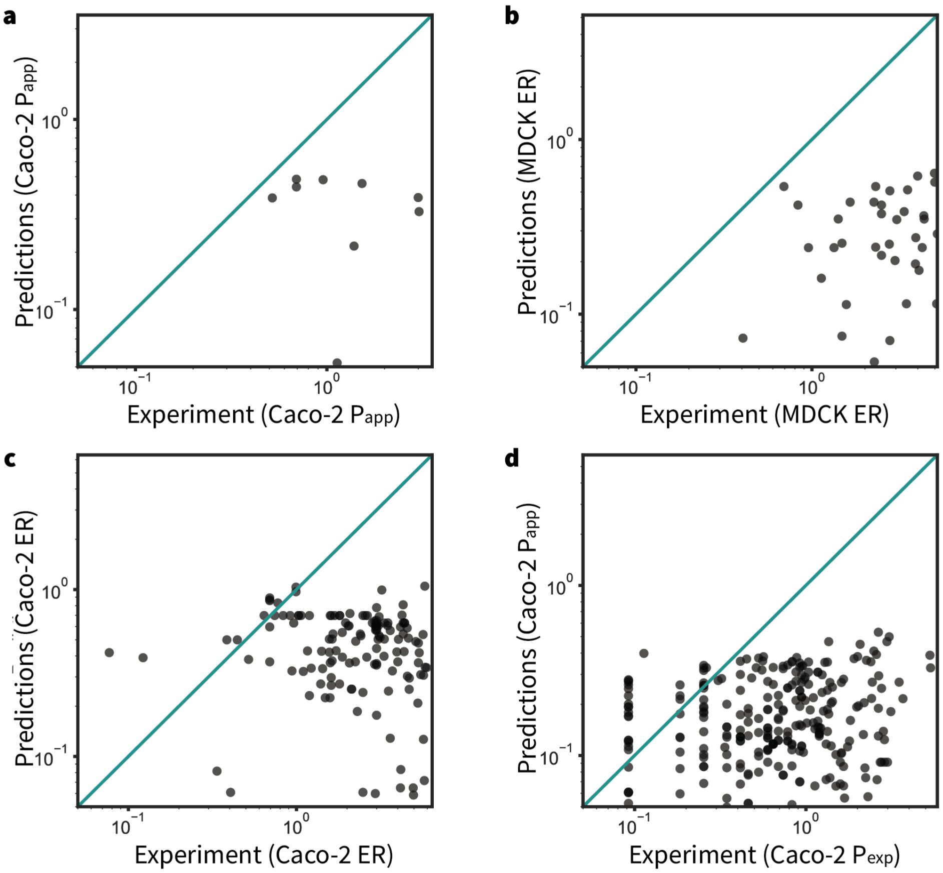

Figure S4: Comparison of predictions made by the GNN-MTL model against public experimental data. a) Predicted Caco-2 versus Caco-2 values from NMMPDB (ρ = 0.40). b) Predicted MDCK ER versus MDCK ER values from NMMPDB (ρ = 0.46). c) Predicted Caco-2 ER versus Caco-2 ER values from NMMPDB (ρ = 0.19). d) Predicted Caco-2 versus Caco-2 values from CycPeptMPDB (ρ = 0.32). ρ: Spearman's rank correlation coefficient. All values are log-scaled.

Additional Tables

In Table S1 we list the detailed performance comparison of different models for Caco-2 endpoints across various data splits, corresponding to Figures 4a– 4d. Then, we list in Table S2 the detailed performance comparison of different models for MDCK-MDR1 and NIH MDCK-MDR1 endpoints across various the data splits, corresponding to Figures 4a–4d. All performances were evaluated using Root Mean Square Error (RMSE) and coefficient of determination (R^2). Finally, in Table S3 we list the number of data points in the internal data for each of the four molecular modalities considered in this work.

Table S1: Performance comparison of different models for Caco-2 endpoints across various data splits, corresponding to Figures 4a–4d. Results for the best-performing model are in bold.

Split	Model	Caco-2 ER		Caco-2	
		RMSE	R^2	RMSE	R^2
random	RF	0.57 ± 0.01	0.57 ± 0.01	0.47 ± 0.01	0.68 ± 0.01
random	MTL	0.55 ± 0.01	0.59 ± 0.01	0.47 ± 0.01	0.69 ± 0.01
random	STL	0.58 ± 0.01	0.56 ± 0.01	0.49 ± 0.02	0.65 ± 0.02
random	MTL+	0.54 ± 0.01	0.62 ± 0.01	0.43 ± 0.01	0.74 ± 0.00
random	MTL++	**0.49 ± 0.01**	**0.68 ± 0.01**	**0.40 ± 0.01**	**0.78 ± 0.01**
scaffold	RF	0.60 ± 0.01	0.50 ± 0.04	0.49 ± 0.02	0.62 ± 0.01
scaffold	MTL	0.55 ± 0.03	0.58 ± 0.03	0.48 ± 0.01	0.68 ± 0.02
scaffold	STL	0.60 ± 0.00	0.50 ± 0.03	0.53 ± 0.03	0.57 ± 0.02
scaffold	MTL+	0.56 ± 0.02	0.59 ± 0.03	0.43 ± 0.00	0.74 ± 0.00
scaffold	MTL++	**0.50 ± 0.01**	**0.66 ± 0.02**	**0.40 ± 0.00**	**0.78 ± 0.01**
leaky	RF	0.59 ± 0.0	0.48 ± 0.0	0.54 ± 0.0	0.61 ± 0.0
leaky	MTL	0.64 ± 0.01	0.40 ± 0.01	0.58 ± 0.01	0.56 ± 0.02
leaky	STL	0.67 ± 0.01	0.34 ± 0.02	0.59 ± 0.01	0.55 ± 0.01
leaky	MTL+	0.60 ± 0.01	0.47 ± 0.03	0.48 ± 0.00	0.70 ± 0.00
leaky	MTL++	**0.54 ± 0.00**	**0.56 ± 0.00**	**0.44 ± 0.01**	**0.75 ± 0.01**
all-for-one	RF	0.59 ± 0.0	0.48 ± 0.0	0.55 ± 0.0	0.61 ± 0.0
all-for-one	MTL	0.65 ± 0.03	0.37 ± 0.05	0.57 ± 0.01	0.58 ± 0.01
all-for-one	STL	0.69 ± 0.02	0.29 ± 0.05	0.59 ± 0.01	0.55 ± 0.02
all-for-one	MTL+	0.61 ± 0.01	0.45 ± 0.02	0.48 ± 0.01	0.70 ± 0.01
all-for-one	MTL++	**0.53 ± 0.01**	**0.58 ± 0.01**	**0.45 ± 0.00**	**0.74 ± 0.00**

Table S2: Performance comparison of different models for MDCK-MDR1 and NIH MDCK endpoints across various data splits, corresponding to Figures 4a-4d. Results for the best-performing model are in bold.

**Split**	**Model**	**MDCK-MDR1**		**NIH MDCK**	
		**RMSE**	**R^2**	**RMSE**	**R^2**
random	RF	0.47 ± 0.01	0.58 ± 0.01	0.34 ± 0.01	0.58 ± 0.03
random	MTL	0.46 ± 0.02	0.59 ± 0.03	0.35 ± 0.06	0.55 ± 0.16
random	STL	0.50 ± 0.01	0.52 ± 0.03	0.38 ± 0.02	0.48 ± 0.03
random	MTL+	0.45 ± 0.01	0.61 ± 0.02	0.32 ± 0.02	0.62 ± 0.03
random	MTL++	**0.42 ± 0.01**	**0.65 ± 0.02**	**0.28 ± 0.01**	**0.71 ± 0.03**
scaffold	RF	0.53 ± 0.05	0.46 ± 0.10	0.37 ± 0.01	0.45 ± 0.01
scaffold	MTL	0.49 ± 0.03	0.59 ± 0.07	0.36 ± 0.04	0.55 ± 0.06
scaffold	STL	0.54 ± 0.05	0.44 ± 0.10	0.40 ± 0.04	0.36 ± 0.12
scaffold	MTL+	0.46 ± 0.02	0.59 ± 0.04	0.35 ± 0.02	0.59 ± 0.07
scaffold	MTL++	**0.44 ± 0.02**	**0.64 ± 0.04**	**0.32 ± 0.03**	**0.66 ± 0.06**
leaky	RF	0.41 ± 0.0	0.38 ± 0.01	0.51 ± 0.0	0.34 ± 0.0
leaky	MTL	0.45 ± 0.02	0.27 ± 0.08	0.47 ± 0.01	0.45 ± 0.03
leaky	STL	0.55 ± 0.03	-0.09 ± 0.12	0.56 ± 0.01	0.22 ± 0.03
leaky	MTL+	0.45 ± 0.03	0.27 ± 0.09	0.47 ± 0.01	0.44 ± 0.03
leaky	MTL++	**0.48 ± 0.01**	**0.19 ± 0.05**	**0.42 ± 0.01**	**0.57 ± 0.03**
all-for-one	RF	0.42 ± 0.0	0.39 ± 0.01	0.45 ± 0.0	0.38 ± 0.01
all-for-one	MTL	0.45 ± 0.02	0.27 ± 0.07	0.42 ± 0.01	0.47 ± 0.03
all-for-one	STL	0.55 ± 0.02	-0.08 ± 0.10	0.50 ± 0.01	0.23 ± 0.02
all-for-one	MTL+	**0.43 ± 0.04**	**0.34 ± 0.14**	0.43 ± 0.01	**0.45 ± 0.04**
all-for-one	MTL++	**0.49 ± 0.03**	**0.17 ± 0.11**	**0.37 ± 0.01**	**0.59 ± 0.03**

Table S3: Number of data points for each endpoint across different molecular categories.

**Endpoint**	**Modality**	**Data Points**
Caco-2	PROTACs	711
Caco-2	Peptides	55
Caco-2	Macrocycles	368
Caco-2	Small molecules	12,755
Caco-2 ER	PROTACs	350
Caco-2 ER	Peptides	50
Caco-2 ER	Macrocycles	259
Caco-2 ER	Small molecules	11,834
MDCK-MDR1 ER	PROTACs	5
MDCK-MDR1 ER	Peptides	18
MDCK-MDR1 ER	Macrocycles	12
MDCK-MDR1 ER	Small molecules	4,767
NIH MDCK ER	PROTACs	3
NIH MDCK ER	Peptides	3
NIH MDCK ER	Macrocycles	1
NIH MDCK ER	Small molecules	3,074
